# Granular cell tumor of the trunk of the facial nerve: A case report: Erratum

**DOI:** 10.1097/MD.0000000000017951

**Published:** 2019-11-11

In the article, “Granular cell tumor of the trunk of the facial nerve: A case report”,^[1]^ which appeared in Volume 98, Issue 19 of *Medicine*¸ Dr. Qian Qiyong's name appears incorrectly as Qiang Qiyong.
